# Drosophila *O*-GlcNAcase Mutants Reveal an Expanded Glycoproteome and Novel Growth and Longevity Phenotypes

**DOI:** 10.3390/cells10051026

**Published:** 2021-04-27

**Authors:** Ilhan Akan, Adnan Halim, Sergey Y. Vakhrushev, Henrik Clausen, John A. Hanover

**Affiliations:** 1 Laboratory of Cell and Molecular Biology, National Institute of Diabetes and Digestive and Kidney Diseases, National Institutes of Health, Bethesda, MD 20892, USA; ilhan.akan@nih.gov; 2 Copenhagen Center for Glycomics, Departments of Cellular and Molecular Medicine and Odontology, Faculty of Health Sciences, University of Copenhagen, DK-2200 Copenhagen, Denmark; halim@sund.ku.dk (A.H.); seva@sund.ku.dk (S.Y.V.); hclau@sund.ku.dk (H.C.)

**Keywords:** *O*-GlcNAc proteome, growth, short life span, HCF, KISMET, SIN3A, FOXO

## Abstract

The reversible posttranslational *O*-GlcNAc modification of serine or threonine residues of intracellular proteins is involved in many cellular events from signaling cascades to epigenetic and transcriptional regulation. *O*-GlcNAcylation is a conserved nutrient-dependent process involving two enzymes, with *O*-GlcNAc transferase (OGT) adding *O*-GlcNAc and with *O*-GlcNAcase (OGA) removing it in a manner that’s protein- and context-dependent. *O*-GlcNAcylation is essential for epigenetic regulation of gene expression through its action on Polycomb and Trithorax and COMPASS complexes. However, the important role of *O*-GlcNAc in adult life and health span has been largely unexplored, mainly due the lack of available model systems. Cataloging the *O*-GlcNAc proteome has proven useful in understanding the biology of this modification in vivo. In this study, we leveraged a recently developed *oga* knockout fly mutant to identify the *O*-GlcNAcylated proteins in adult *Drosophila* *melanogaster*. The adult *O*-GlcNAc proteome revealed many proteins related to cell and organismal growth, development, differentiation, and epigenetics. We identified many *O*-GlcNAcylated proteins that play a role in increased growth and decreased longevity, including HCF, SIN3A, LOLA, KISMET, ATX2, SHOT, and FOXO. Interestingly, *oga* mutant flies are larger and have a shorter life span compared to wild type flies, suggesting increased *O*-GlcNAc results in increased growth. Our results suggest that *O*-GlcNAc alters the function of many proteins related to transcription, epigenetic modification and signaling pathways that regulate growth rate and longevity. Therefore, our findings highlight the importance of *O*-GlcNAc in growth and life span in adult *Drosophila*.

## 1. Introduction

Growth is closely dependent on nutrient availability. Many growth-related pathways are regulated by the phosphorylation/dephosphorylation of serine/threonine (Ser/Thr) residues by numerous kinases and phosphatases, respectively. The same or similar Ser/Thr sites on a given intracellular protein can also be modified by N-acetylglucosamine (GlcNAc) [1]. *O*-GlcNAcylation could alter the phosphorylation of a particular site, thereby regulating the activity of the target protein [2,3,4]. The enzyme in charge of adding GlcNAc is *O*-GlcNAc transferase (OGT), while the enzyme cleaving it is *O*-GlcNAcase (OGA), in a dynamic fashion. The synthesis of GlcNAc requires intracellular nutrition-derived molecules, including glucose, glutamine, and acetyl Co-A, making this molecule and the enzymes using it susceptible to nutrient status. Therefore, *O*-GlcNAcylation is termed as an intracellular nutrient sensor connecting the nutrition-to-cellular pathways from signaling to transcription and epigenetics [5].

*O*-GlcNAc came into the spotlight through the studies performed in *Drosophila melanogaster*, which showed that OGT/sxc is a member of the epigenetic repressor family called the Polycomb repressor complex [6]. Indeed, the *O*-GlcNAcylation of Polyhomeotic (Ph) is essential for Polycomb (Pc) complex-dependent repression of Hox genes [7,8]. In addition, we recently showed that gene activation through Trithorax and COMPASS complexes is also regulated by *O*-GlcNAcylation [9]. Therefore, the nutrient sensor *O*-GlcNAc seems to regulate growth through both the repression and activation of gene expression in a context-dependent manner.

Thousands of mammalian proteins are thought to be *O*-GlcNAcylated [10,11,12,13]. The vast number of proteins in addition to the redundancies between mammalian protein isoforms create major barriers in studying, and thereby understanding, the biology of protein *O*-GlcNAcylation [14]. The use of simpler model organisms with fewer *O*-GlcNAcylated proteins that could be genetically targeted and studied in vivo would significantly increase our knowledge of the role of *O*-GlcNAcylation in health and disease. One such organism, *Drosophila melanogaster,* has been used to detect the *O*-GlcNAcylated proteins in early development and embryogenesis [15,16], which has led to the discovery of a limited number of *O*-GlcNAcylated proteins [15,16]. To complement these studies, there is a need for establishing the *O*-GlcNAc proteome in adult *Drosophila* that could serve as a database related to chronic diseases.

*O*-GlcNAcylation is implicated in chronic late onset diseases, such as type II diabetes and Alzheimer’s [11,12,17,18]. Interestingly, *Drosophila* has long served as a suitable model organism for diabetes and neurodegenerative diseases [11,12,17,18]. Therefore, we decided to use adult flies to detect *O*-GlcNAcylated proteins that are potentially important during adulthood and could be important players in chronic diseases. We elected to use the previously generated *oga* mutant flies (*oga^del.1^*) to perform a proteomics study that would not be plagued by *O*-GlcNAc removal during isolation and define those sites where *O*-GlcNAc turnover might normally occur [9]. Here, we report that 102 proteins in females and 53 proteins in males are detected as *O*-GlcNAcylated in adult flies, and 42 proteins were common to both sexes. These common 42 proteins were related to growth and organism development. Upon carefully examining the phenotype of *oga^del.1^* mutant flies, we found *oga* mutants indeed were larger than wild type flies. In addition, the *oga* mutants displayed shorter life spans compared to wild type flies. Moreover, among the identified proteins in the *O*-GlcNAc proteome, a chromatin modifier protein, Trithorax-related (TRR), was shown to co-localize with *O*-GlcNAc in a subset of genomic regions on polytene chromosomes.

Our study is the first to systematically characterize *O*-GlcNAcylated proteins in adult *Drosophila*. The findings significantly increased the number of *O*-GlcNAcylated proteins in an adult model organism that is amenable to genetic manipulation. Consistent with the *O*-GlcNAc proteome results, we showed that the *oga^del.1^* mutants display increased body size and shortened life spans. The data presented here suggest that *Drosophila* has promise as a useful model organism for the role of *O*-GlcNAc turnover in chronic diseases like diabetes and neurodegeneration.

## 2. Materials and Methods

### 2.1. Fly Stocks

The 13618 OGA P element insertion, *ogt/sxc* mutants, Actin-Gal4, esg-GAL4, and the deficiency line spanning *oga* gene B9485 were from the Bloomington Stock Center. The UAS-OGA-RNAi fly line was obtained from VDRC [19]. The reported UAS-OGA overexpression lines were originally generated by Kaasik et al. [20]. The *oga^del.1^* mutant was generated by standard P-element excision protocol [21]. Flies were maintained at 25 °C in a humidified incubator. Drosophila MM media was purchased from KD medical (Columbia, MD, USA).

### 2.2. Longevity Assay

Life span analysis was performed as described previously [22]. Flies were raised to adulthood at 25 °C. The 1–2-day-old flies were placed in vials (20 flies per vial). Males and females were kept in separate vials. Dead flies were monitored daily, and the vials were changed every 3 days. The experiment continued until all the flies were dead. Survival curves were generated by calculating the percentage of surviving flies and plotting this as a function of time in days.

### 2.3. Wing Size Measurement

Wing size measurement was performed as described previously [23]. Newly hatched flies were briefly transferred to fresh fool vials and kept at 25 °C until they reached the age of 8 days. Wings were dissected and digitally photographed using a Zeiss camera. The pictures were printed and the distance between the L4 and L5 veins was measured.

### 2.4. Polytene Chromosome Staining and Imaging

Polytene chromosomes were prepared as described previously [24]. For immunostaining, the slides containing polytenes were incubated with 100%, 50% and 25% ethanol followed by PBSTx (0.1% TRX 100). After 3 washes with PBSTx, the slides were blocked with Odyssey blocking reagent for 1 h at room temperature and incubated with rabbit-anti-TRR antibody at 1/50 dilution, along with *O*-GlcNAc-specific antibody at 1/100 dilution overnight at 4 °C in a humidified chamber. The next day, the slides were washed 4 times with PBSTx and incubated with Alexa Fluor-conjugated secondary antibodies in the dark for 2 h at room temperature. The slides were then mounted in a Slowfade mounting medium (Invitrogen, Carlsbad, CA, USA) and visualized using Zeiss LSM 700 confocal microscope with Zen imaging software (Zeiss, Oberkochen, Germany). Antibodies: anti *O*-GlcNAc (#MA1-076, Thermo Fisher Scientific, St Peters, MO, USA), rabbit anti-TRR was a kind gift from Dr. Shilatifard [25]. All secondary antibodies were Alexa Fluor 488- or Alexa Fluor 568-conjugated (Invitrogen) and used at 1/250 dilution.

### 2.5. Mass Spectrometry

Both male and female flies were prepared at 4 °C by homogenization in 50 mM ammonium bicarbonate, 0.05% Rapigest (Waters, Milford, MA, USA) buffer. Following a brief (30 min) incubation on ice, each sample was sonicated before the removal of tissue/cell debris by centrifugation (2000× *g*, 4 °C, 10 min). Total protein extracts were reduced by 5 mM dithiothreitol (60 °C, 30 min) and alkylated with 10 mM iodoacetamide at room temperature (RT) in the dark for 30 min before digestion with 25 µg trypsin (37 °C, 16 h). Tryptic peptides were labeled with dimethyl stable isotopes [26] using a light reagent for WT and a medium (deuterated) reagent for *oga^del.1^* knockout samples. WT and *oga^del.1^* knockout tryptic digests were subsequently mixed at a 1:1 ratio and desalted on Sep-Pak C18 cartridges (Waters). The tryptic digests were then treated with PNGase F and PNGase A glycosidases to remove *N*-linked (GlcNAc) glycans. The mixed and labeled sample was passed through a 2.8 m ConA column as previously described [27], mainly to enrich for *O*-Man glycopeptides but also to remove any remaining *N*-glycopeptides. The flow-through fraction from the ConA was then passed through a VVA column for the depletion of *O*-GalNAc glycopeptides. Finally, the VVA flow-through fraction was enriched using an in-house packed 10 m WGA-agarose (Vector Laboratories, Burlingame, CA, USA) column. Five percent (*v*/*v*) of each flow-through, wash and elution (0.5 M GlcNAc) fraction was screened by mass spectrometry for *m*/*z* 204 oxonium ions [28]. Selected fractions were then pooled, concentrated, and subjected to a second round of WGA chromatography. For female flies, fractions containing *O*-GlcNAc were once again pooled, purified by C18 reversed phase chromatography and further fractionated by isoelectric focusing into 12 fractions, as previously described [27]. For male flies, *O*-GlcNAc peptides were enriched by sequential WGA chromatography before high-pH reversed-phase orthogonal fractionation into 8 fractions. IEF and high-pH fractions were analyzed on a Orbitrap Fusion mass spectrometer as previously described [29]. Briefly, each fraction was separated using an analytical column packed in-house with Reprosil-Pure-AQ C18 phase (120 min gradient) and analyzed by data-dependent acquisition. Multiply charged precursor ions were selected for HCD and ETciD fragmentation. Data processing was carried out using Proteome Discoverer 1.4. Raw files were processed using MS Amanda/Sequest HT nodes and searched against the canonical D. melanogaster proteome from Uniprot (January, 2016). Carbamidomethyl (Cys: +57.021 Da) and dimethyl (peptide N-term and Lys: +28.031 Da or +32.056 Da) were set as static modifications. Oxidation (Met: +15.995 Da) and HexNAc (Ser/Thr: +203.079 Da) were set as variable modifications. One missed tryptic (full- and semi-specific) cleavage was allowed. The precursor ion tolerance was <10 ppm and MS2 fragment tolerance was set to 0.02 Da. The false discovery rate was calculated by the Target Decoy PSM validator note and only highly confident peptide hits were considered. The identified PSMs were further validated by manual inspection to assure correct assignments. MS1-level quantification was performed using the Precursor Ions Quantifier node. The mass spectrometry proteomics data have been deposited into the ProteomeXchange Consortium via the PRIDE [30] partner repository with the dataset identifier PXD025344.

## 3. Results

### 3.1. Adult O-GlcNAc Proteome Revealed Growth and Development Related Proteins

**Figure 1:**
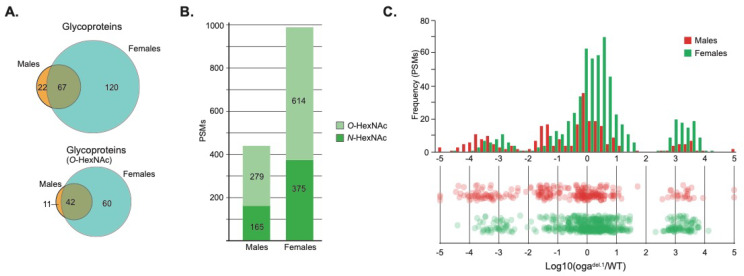
Differential *O*-GlcNAc glycoproteomics of *oga^del.1^* male and female flies. (**A**) Venn diagrams of total glycoproteins (top) and *O*-HexNAc glycoproteins (bottom) identified by mass spectrometry; (**B**) stacked bar-chart showing the number of peptide spectral matches for *N*-linked and *O*-linked glycopeptides identified in both sexes; (**C**) quantification of relative abundances of *O*-GlcNAcylation between wild type and *oga^del.1^* male and female flies. Frequency bars (top) show the number of PSMs plotted against log10 transformed medium-to-light ratios (*oga^del.1^*/WT). The distribution of medium-to-light ratio datapoints is also presented as a dot-plot (bottom).

We examined *O*-GlcNAcylated proteins in adulthood using 8-day-old flies. The identification of *O*-GlcNAc glycopeptides and the quantification of relative changes in the *O*-GlcNAc status were achieved by differential glycoproteomics. Wild type and *oga^del.1^* mutant proteomes were isotopically labeled with dimethyl stable isotopes and mixed before enrichment by sequential lectin weak affinity chromatography (LWAC). Isotope-labeled tryptic digests were then passed through a Concanavalin A (ConA) and a Vicia Villosa (VVA) column before the flow-through fraction was enriched by wheat germ agglutinin (WGA) LWAC. We rationalized that this approach would deplete high-mannose-type *N*-glycopeptides and *O*-GalNAc-type glycopeptides from the tryptic digest before the *O*-GlcNAc glycopeptides could be enriched by WGA-LWAC. Finally, selected WGA-LWAC fractions were pooled and further fractionated by isoelectric focusing (females) or high-pH fractionation (males), before characterization by high-resolution mass spectrometry utilizing higher-energy collisional dissociation (HCD) and hybrid electron transfer dissociation (ETciD) fragmentation modes. This approach allowed us to identify 187 and 89 HexNAc-modified proteins in female and male flies, respectively (Figure 1A, Supplementary Table S1). The female and male datasets were based on 989 and 444 peptide spectral matches (PSMs), respectively (Figure 1B). All PSMs were manually inspected to confirm fragment ion assignments and representative MS2 spectra are shown in Supplementary Figure S1. In both datasets, we identified a number of glycopeptide sequences containing the NX(S/T) consensus sequence for *N*-glycosylation, originating from proteins that are expected to traffic the secretory pathway, e.g., collagen, integrins and laminin. Upon closer inspection, HCD/EtciD fragmentation clearly demonstrated that asparagine (Asn), and not serine (Ser) or threonine (Thr) residues, were modified by HexNAc glycans (Supplementary Table S1). We therefore excluded all glycopeptides containing the NX(S/T) consensus sequence from further analysis, which reduced the number of *O*-HexNAc glycoprotein identifications to 53 and 102 for male and female flies, respectively (Figure 1A). We observed that 42 glycoproteins were common to both sexes. GO term enrichment of these 42 proteins revealed the enrichment of biological processes related to growth, such as cell development, organ development, and anatomical structure development (Table 1). The presence of factors implicated in imaginal disc development, organelle organization and cell development, such as SIN3A, KIS, LOLA, HCF, TRR, and ENOK (Supplementary Figure S1), is of particular interest as they are suggested to play roles in both chromatin and neuronal maintenance. Of note, all the *O*-GlcNAc-modified sites that were detected on HCF are different from the sites reported previously [16]. Whether these differences are biologically relevant remains to be studied.

The isotopically encoded precursor ion profiles in MS^1^ were used to compare the abundance of *O*-GlcNAcylations between wild type and *oga^del.1^* mutant fly extracts (Figure 1C). For females, we observed 40 glycoproteins with increased (>10-fold) and 24 glycoproteins with decreased (>10-fold) *O*-GlcNAcylation as a function of *oga^del.1^* (Figure 1C and Supplementary Table S1). Males displayed a similar pattern, with 14 and 12 glycoproteins showing a relative increase or decrease in *O*-GlcNAcylation, respectively. For both sexes, we also observed that individual glycoproteins had both increased and decreased *O*-GlcNAcylation at different sites. The identification of specific proteins with altered *O*-GlcNAc abundances therefore suggests that *O*-GlcNAcylation is protein- and context-dependent. Perhaps for certain proteins/sites, *O*-GlcNAcylation by OGT is important, while other proteins/sites require the removal of OGA for their function. Taken together, the effect of *O*-GlcNAc on every protein seems rather unique to the individual protein, or even each *O*-GlcNAcylated site. This kind of unique regulation could be important for the function of proteins such as HCF, KIS, and TRR, which play key roles in gene expression [31,32,33,34,35].

### 3.2. oga^del.1^ Flies Have Larger Body Size Than Wild Type Flies

**Figure 2:**
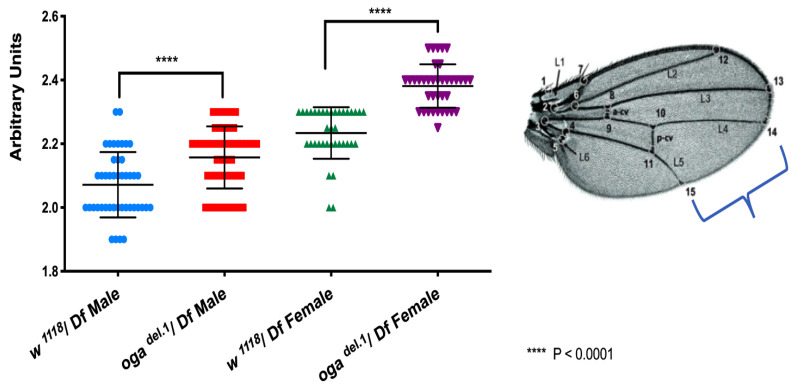
*oga^del.1^* mutants have increased body size. The wings of 8-day-old adult flies that have been kept at 25 °C were used for this assay. The distance between L4 and L5 wing veins was measured. Both female and male *oga^del.1^* mutants were larger than wild type flies. Statistical analysis was performed using unpaired *t*-test. **** = *p* < 0001.

Motivated by the fact that proteins that are common to both females and males were related to growth (Table 1), we compared the body sizes of the *oga^del.1^* mutant versus wild type flies. Wild type and *oga^del.1^* mutants were crossed with an *oga* deficiency fly line to eliminate possible secondary mutations that might account for the phenotype. We measured the distance between L4 and L5 wing veins, as wings are known to be the only organs that do not change in size due to diet in adult flies. Indeed, both female and male *oga^del.1^* mutants were significantly larger than wild type flies (Figure 2), emphasizing the importance of protein *O*-GlcNAcylation in growth.

### 3.3. O-GlcNAc Levels Are Important for Imaginal Wing Disc

**Figure 3:**
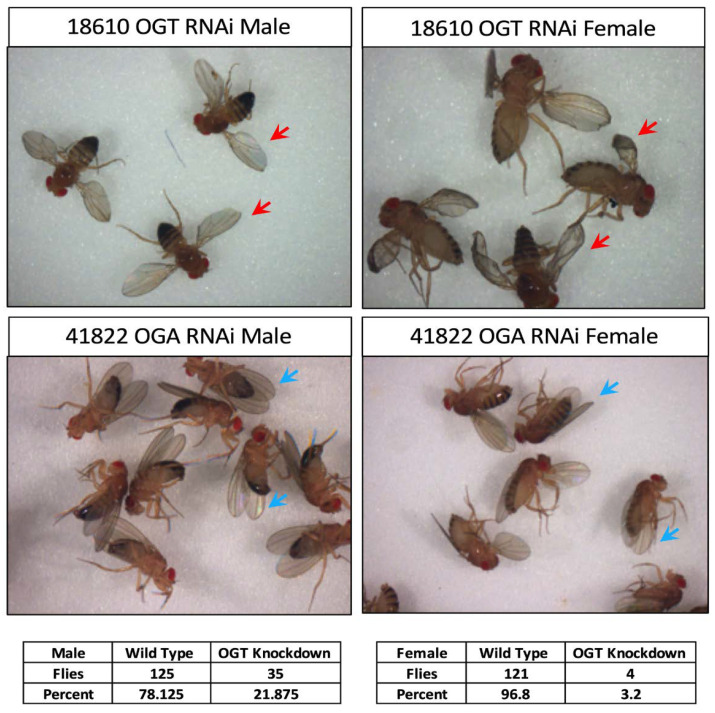
Knockdown of OGT in wing imaginal disc causes a strong phenotype in females. OGT knockdown in wing imaginal discs causes lethality (3% survival, compared to 25% expected) along with wing development defects in females. OGT knockdown in wing imaginal of males did not cause lethality (~22% survival, 25% expected), and males only showed the wings-down phenotype. In comparison, OGA knockdown has no effect on survival and wing development.

Development was seen among the GO term-enriched groups in our proteomics dataset. We therefore decided to assess the importance of *O*-GlcNAc using an observable phenotype. Hence, we chose to observe wings. We were particularly interested in whether increase or decrease in *O*-GlcNAc levels had any effect on wing development. Since the knockout of *ogt* is lethal in flies, we decided to alter the gene expression using RNAi. *O*-GlcNAc levels were decreased by OGT RNAi and increased by OGA RNAi using esg-Gal4 driver, which is important for wing development. The knockdown of OGT in cells expressing esg-Gal4 had severe wing development effects and resulted in lethality in females (3% survival as opposed to 25% expected). In addition, the decrease in *O*-GlcNAc levels by OGT RNAi caused strong wing development defects in surviving females (Figure 3). Interestingly, males showed a wings-down phenotype, but no other wing development defect (Figure 3). In contrast, the increased *O*-GlcNAc by OGA RNAi had no observable phenotype in wing development. In summary, the results suggest that a decrease in *O*-GlcNAcylated protein levels are not tolerated during development, while an increase in *O*-GlcNAcylation has no effect.

### 3.4. oga^del.1^ Flies Display Shorter Life Span Compared to Wild Type Flies

**Figure 4:**
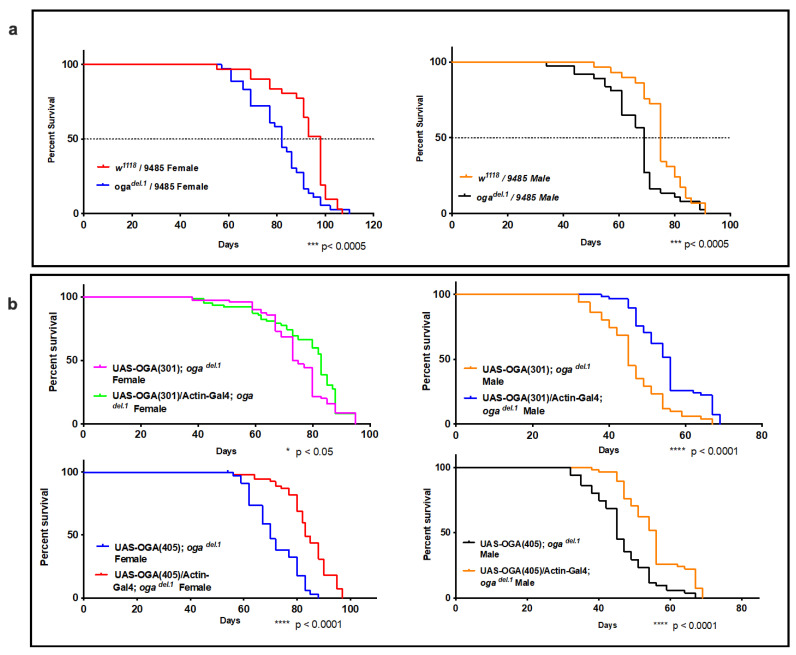
(**a**) *oga^del.1^* mutants have short life spans. Both male and female *oga^del.1^* mutant flies display shorter life spans compared to wild type flies; (**b**) ectopic expression of OGA rescued the short life span of *oga^del.1^* mutants. Statistical comparisons were performed via log-rank test. **** = *p* < 0.0001, *** *p* < 0.0005, * *p* < 0.05.

An increase in body size could cause problems in animal health and life span. Therefore, we decided to determine whether longevity is affected in *oga^del.1^* mutants. As suspected, we observed a significant decrease in the longevity of both female and male *oga^del.1^* flies (Figure 4a). The average lifespan of *oga^del.1^* mutants was about 10 days shorter (a ~15% decrease) than the wild type counterparts. In order to confirm that the shortened life span was due to the loss of the *oga* gene, we ectopically expressed OGA under the control of the actin promoter. The ectopic expression of OGA rescued the longevity defect of *oga^del.1^* mutant flies (Figure 4b). Collectively, *oga^del.1^* mutant flies have bigger bodies and a shorter life span.

### 3.5. TRR Is Co-Stained with O-GlcNAc on Polytene Chromosomes

**Figure 5:**
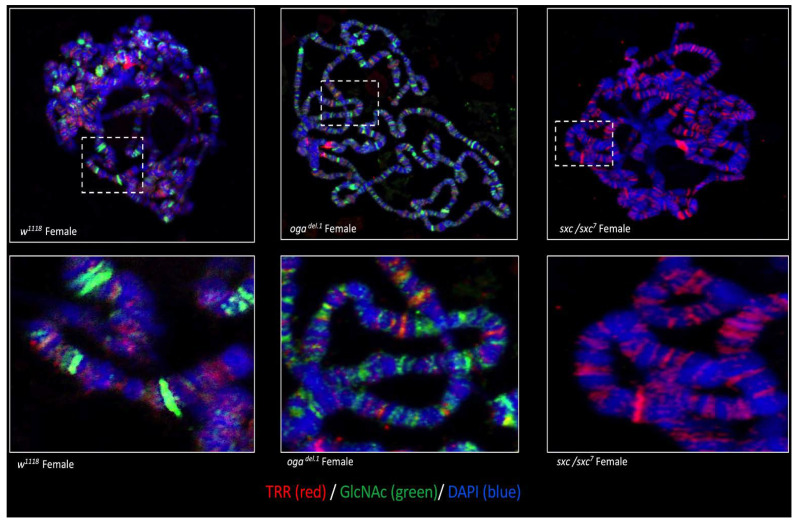
Trithorax-related (TRR) colocalizes with *O*-GlcNAc in certain genomic regions on the polytene chromosomes. Polytene chromosomes prepared from the third instar larvae of wild type (*w^1118^*), oga mutants (*oga^del.1^*) and ogt mutant (*sxc^1^/sxc^7^*) were stained for TRR (red), *O*-GlcNAc (green), and DAPI (DNA). TRR co-stained with *O*-GlcNAc on certain genomic regions. Polytenes from *sxc^1^/sxc^7^* were used as a negative control for O-GlcNAc staining.

TRR is an epigenetic activator of gene expression, which was detected in our proteomics data (Supplementary Table S1). We were interested in whether the *O*-GlcNAcylation of TRR is global, or specific to certain genomic regions. Based on our previous experience with a different protein [9], we predicted that not all the TRR-regulated genomic regions would be positive for *O*-GlcNAc. To observe the co-localization, polytene chromosomes from female third instar larva were prepared (Figure 5). As suspected, TRR showed colocalization with *O*-GlcNAc on the polytene chromosomes at certain genomic regions, but not all the TRR-positive sites were also stained positive for *O*-GlcNAc, suggesting a gene/context-specific regulation.

In summary, we showed that *O*-GlcNAc is essential for regulating the growth rate in adult Drosophila. Increased *O*-GlcNAc levels in the absence of OGA lead to increased organism size and a short life span. We cataloged many proteins that could potentially play a role in this phenotype. Our results also suggest that *O*-GlcNAc regulation is context-dependent, meaning both the addition of *O*-GlcNAc by OGT and the removal of *O*-GlcNAc by OGA of a particular protein/site are important for their function.

## 4. Discussion

The regulation of protein *O*-GlcNAcylation during adulthood is an important yet understudied area. The only model organism that survives without a functioning *O*-GlcNAc OGT/OGA cycle is *C. elegans,* referring to a viable nematode lacking both *Ogt* and *Oga* enzymes [36]. Our lab showed that the loss of *Oga* increased the phenotype of neurodegenerative disease models, while the loss of *Ogt* averted this phenotype [36] in *C. elegans*. In contrast to worms, *Drosophila* melanogaster cannot survive without *ogt* [6,8]. However, we were able to generate viable *oga* mutant flies [9]. The use of adult *oga^del.1^* in this study underlined the importance of *O*-GlcNAc turnover and oga to maintain physiological levels of *O*-GlcNAc for longevity. Strikingly, the *oga^del.1^* mutant flies displayed shortened life spans (Figure 4), a phenotype frequently observed in neurodegeneration [37,38,39]. While in agreement with our previous finding that the loss of *oga* enhanced the phenotype of neurodegenerative disease models, the molecular reasons behind the longevity defect in *oga^del.1^* flies are currently unknown. Proteins related to neurogenesis, axon guidance, and chromatin remodeling, such as LOLA, ATX2, KIS, HCF, SIN3A, and ENOK, were enriched in our *O*-GlcNAc proteome dataset. Elucidating the mechanisms and the proteins involved in the longevity defect phenotype will provide invaluable information. Our study can provide critical insight about the role of *O-*GlcNAc in neural maintenance, since inhibitors of OGA are being studied as potential drugs for neurodegenerative disease models [40,41].

### 4.1. Oga Mutant Flies Facilitates Glycoproteomics

The ability to produce enough mass for proteomic experiments allowed us to determine the *O*-GlcNAc proteome using *oga^del.1^* mutant adult flies. We greatly increased the number of known *O*-GlcNAcylated proteins in *Drosophila*, paving the way for more studies to come. The availability of *oga^del.1^* flies will increase the pace of future research on fly models associated with *O*-GlcNAc and chronic disease. Moreover, the separation of males from females suggests possible gender differences in protein *O-*GlcNAcylation. Interestingly, the reduced *O*-GlcNAcylation in wing imaginal discs displayed a stronger phenotype in females. Clarifying a possible gender difference in *O*-GlcNAcylation will be very interesting.

### 4.2. Glycoproteomics Reveals Novel O-GlcNAc Modified Proteins Related to Growth and Longevity

The major group of proteins that are detected in the adult *O*-GlcNAc proteome are related to growth (Table 1). Indeed, in agreement with the proteomics results, both female and male *oga^del.1^* mutant flies are larger than the wild type flies (Figure 2). Intriguingly, the basic region of the HCF protein, which interacts with SIN3A, is suggested to regulate cell division [42].The *O*-GlcNAcylated Ser/Thr sites that we described in this study reside within the aforementioned basic region. It is intriguing to speculate that *O*-GlcNAcylation might in part regulate cell division through modifying the HCF basic region. Moreover, many of the other proteins we detected in the proteomics screen could play a role in this phenotype. Sin3A is an important epigenetic factor for stem cells [43,44]. Together with its complex partners, including HCF, Sin3A could be involved in increased body size in *oga* mutant flies. Epigenetic factors such as ENOK and STWL, which have been reported to be important for germline stem cells, could be involved as well [45,46]. In addition, signaling proteins such as FOXO could increase cell division, and thereby body size [47,48].

The altered function of FOXO is suggested to affect longevity [49,50]. In light of these findings, our results suggest that the non-cyclical *O*-GlcNAc modification of FOXO, at least in part, could lead to organism overgrowth and negatively impact longevity. Interestingly, we have shown previously that *O*-GlcNAc occupies chromatin related to longevity in C. elegans [51]. There are many chromatin-modifying and-remodeling proteins, such as HCF, SIN3A, E(bx), ENOK, KIS, TRR, and STWL, in our *Drosophila O*-GlcNAc proteome. Among the identified proteins, HCF and Sin3A have both been shown to impact longevity [52,53,54]. One of the chromatin-related proteins we discovered, TRR, colocalizes with *O*-GlcNAc on a subset of their targets on polytene chromosomes (Figure 5). It will be interesting to see whether any of the chromatin-associated factors we discovered occupy genomic regions associated with longevity in *Drosophila*. It is feasible that the altered functions of one or more of these factors cause more broad effects on longevity, cell growth, and proliferation.

This study highlights the importance of *O*-GlcNAc on longevity and growth rate, which will have implications for diseases such as neurodegenerative disorders, diabetes and cancer. Because flies are one of the best genetically characterized model organisms, our fly model along with newly discovered *O*-GlcNAcylated proteins in adult flies will be very useful in elucidating the mechanisms by which *O*-GlcNAc impacts growth and longevity.

## Figures and Tables

**Table 1:** Gene ontology analysis of *O*-GlcNAcylated proteins found in both males and females.

GO Term	*p*-Value
positive regulation of gene expression [GO:0010628]	1.06 × 10^−4^
biological regulation [GO:0065007]	1.68 × 10^−4^
positive regulation of cellular process [GO:0048522]	1.95 × 10^−4^
regulation of cellular process [GO:0050794]	3.71 × 10^−4^
regulation of biological process [GO:0050789]	3.94 × 10^−4^
positive regulation of biological process [GO:0048518]	8.27 × 10^−4^
positive regulation of macromolecule biosynthetic process [GO:0010557]	0.001605
positive regulation of macromolecule metabolic process [GO:0010604]	0.001687
growth [GO:0040007]	0.003118
regulation of gene expression [GO:0010468]	0.003782
positive regulation of cellular biosynthetic process [GO:0031328]	0.00389
positive regulation of biosynthetic process [GO:0009891]	0.003967
positive regulation of metabolic process [GO:0009893]	0.005195
cellular component organization [GO:0016043]	0.007664
regulation of cellular macromolecule biosynthetic process [GO:2000112]	0.022871
cellular component organization or biogenesis [GO:0071840]	0.023153
regulation of macromolecule biosynthetic process [GO:0010556]	0.02408
anatomical structure development [GO:0048856]	0.02648
positive regulation of nitrogen compound metabolic process [GO:0051173]	0.027266
developmental process [GO:0032502]	0.029356
neuron development [GO:0048666]	0.032766

## Data Availability

The mass spectrometry proteomics data have been deposited into the ProteomeXchange Consortium via the PRIDE [30] partner repository with the dataset identifier PXD025344.

## Supplementary Materials

The following are available online at https://www.mdpi.com/article/10.3390/cells10051026/s1, Figure S1: Mass spectrometry signature of Select Proteins. Table S1: List of *O*-GlcNAc modified proteins.
