# Supplementary material for: Drosophila O-GlcNAcase Mutants Reveal an Expanded Glycoproteome and Novel Growth and Longevity Phenotypes
Source: Cells. 2021 Apr 27;10(5):1026. doi: 10.3390/cells10051026 (PMC8145559; doi:10.3390/cells10051026)
Supplement: Supplementary file 1 [file cells-10-01026-s001.zip › Proteome Dataset Access Instructions.pdf]

Proteome Dataset Access Details

Dear Adnan Halim,

We are happy to inform you that your dataset "Fruit fly O-GlcNAc glycoproteomics" has been successfully submitted to ProteomeXchange via the PRIDE database. The data is currently private, and can only be accessed with your account or with a single reviewer account that has been created.

Please note that it is essential that you notify us of the first (online) publication of the corresponding manuscript.Otherwise the data will remain inaccessible to readers.

Please add to your manuscript the following sentence (typically in the "Methods" section or just before/in the Acknowledgements):

"The mass spectrometry proteomics data have been deposited to the ProteomeXchange Consortium via the PRIDE [1] partner repository with the dataset identifier PXD025344".

We would recommend you to also include this information in a much abridged form into the abstract itself, e.g. "Data are available via ProteomeXchange with identifier PXD025344."

Submission details:

Project Name: Fruit fly O-GlcNAc glycoproteomics
Project accession: PXD025344
Project DOI: Not applicable

Reviewer account details:

Username: reviewer_pxd025344@ebi.ac.uk
Password: QHWwgkOA

Don't forget to include these reviewer account details in your manuscript, during the peer review process.
