# Supplementary material for: Drosophila O-GlcNAcase Mutants Reveal an Expanded Glycoproteome and Novel Growth and Longevity Phenotypes
Source: Cells. 2021 Apr 27;10(5):1026. doi: 10.3390/cells10051026 (PMC8145559; doi:10.3390/cells10051026)
Supplement: Supplementary file 1 [file cells-10-01026-s001.zip › Supplemental Figure 1.pdf]

Supplemental figure 1. Representative MS/MS spectra of selected O-GlcNAc glycopeptides. The table below details peptide sequences, protein, modifications and additional details related to the annotated spectra. The spectral number above each annotated MS/MS image is linked to the table below.

Sequence	Protein	Activation Type	Modifications	Charge	Precursor m/z [Da]	Spectra no.
**SNSATVPTGTAATAGGATGTTPVVK**	ATX2	EtciD	N-Term(Dimethyl:2H(4)); S1(HexNAc); K25(Dimethyl:2H(4))	3	828.78149	1
**MPSTSGSNTGTSTSSSK**	KISMET	EtciD	N-Term(Dimethyl); S5(HexNAc); K17(Dimethyl)	2	938.42804	2
**QPTSSTTTSIGGK**	HCF	EtciD	N-Term(Dimethyl); S4(HexNAc); K13(Dimethyl)	2	762.39398	3
**NLLNGATSSASNTSSTQSK**	KISMET	EtciD	N-Term(Dimethyl:2H(4)); S8(HexNAc); S9(HexNAc); K19(Dimethyl:2H(4))	3	780.06238	4
**TPAAPTPTSNSATVPTGTAATAGGATGTTPVVK**	ATX2	EtciD	N-Term(Dimethyl); S9(HexNAc); K33(Dimethyl)	4	803.91779	5
**VTSSQTITTITTTVK**	SHOT	EtciD	N-Term(Dimethyl:2H(4)); T2(HexNAc); K15(Dimethyl:2H(4))	3	616.69452	6
**VTSSQTITTITTTVK**	SHOT	HCD	N-Term(Dimethyl:2H(4)); T2(HexNAc); K15(Dimethyl:2H(4))	2	924.53668	7
**GNQQIIIVTTGGNVR**	HCF	HCD	N-Term(Dimethyl:2H(4)); 2x(HexNAc)	2	1004.54675	8
**SSECELINIK**	LOLA	HCD	N-Term(Dimethyl:2H(4)); 1x(HexNAc); C4(Carbamidomethyl); K10(Dimethyl:2H(4))	2	730.39465	9
**TTNAATLTPA**	SINA3	HCD	N-Term(Dimethyl:2H(4)); 1x(HexNAc)	2	598.32184	10
**TTNAATLTPAAGAGAAAA**	SINA3	HCD	N-Term(Dimethyl); 1x(HexNAc)	2	866.44208	11
**SQPSVVTSPPSWVH**	FOXO	HCD	N-Term(Dimethyl:2H(4)); 1x(HexNAc)	2	871.94885	12

No. 1

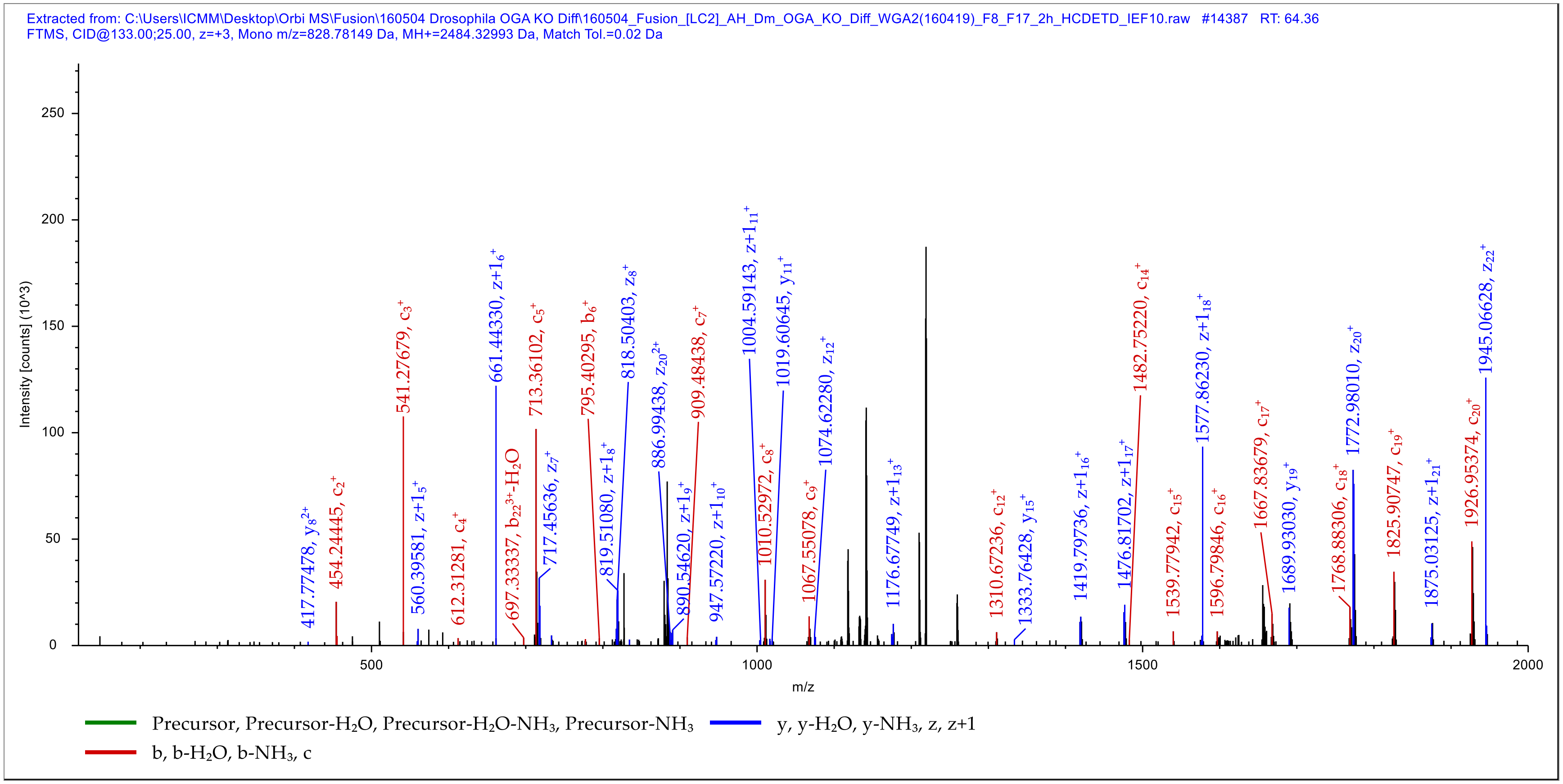
Extracted from: C:\Users\ICMM\Desktop\Orbi MS\Fusion\160504 Drosophila OGA KO Diff\160504_Fusion_[LC2]_AH_Dm_OGA_KO_Diff_WGA2(160419)_F8_F17_2h_HCDETD_IEF10.raw #14387 RT: 64.36
FTMS, CID@133.00;25.00, z=+3, Mono m/z=828.78149 Da, MH+=2484.32993 Da, Match Tol.=0.02 Da
Intensity [counts] (10^3)
0
50
100
150
200
250
500
1000
1500
2000
m/z
417.77478, y_8^{2+}
454.24445, c_2^+
541.27679, c_3^+
560.39581, $z+1_5^+$
612.31281, c_4^+
661.44330, $z+1_6^+$
697.33337, b_{22}^{3+}-H_2O
713.36102, c_5^+
717.45636, z_7^+
795.40295, b_6^+
819.51080, $z+1_8^+$
818.50403, z_8^+
886.99438, z_{20}^{2+}
890.54620, $z+1_9^+$
909.48438, c_7^+
947.57220, $z+1_{10}^+$
1004.59143, $z+1_{11}^+$
1010.52972, c_8^+
1019.60645, y_{11}^+
1067.55078, c_9^+
1074.62280, z_{12}^+
1176.67749, $z+1_{13}^+$
1310.67236, c_{12}^+
1333.76428, y_{15}^+
1419.79736, $z+1_{16}^+$
1476.81702, $z+1_{17}^+$
1482.75220, c_{14}^+
1539.77942, c_{15}^+
1577.86230, $z+1_{18}^+$
1596.79846, c_{16}^+
1667.83679, c_{17}^+
1689.93030, y_{19}^+
1768.88306, c_{18}^+
1772.98010, z_{20}^+
1825.90747, c_{19}^+
1875.03125, $z+1_{21}^+$
1926.95374, c_{20}^+
1945.06628, z_{22}^+
Precursor, Precursor-H_2O, Precursor-H_2O-NH_3, Precursor-NH_3
y, y-H_2O, y-NH_3, z, z+1
b, b-H_2O, b-NH_3, c

No. 2

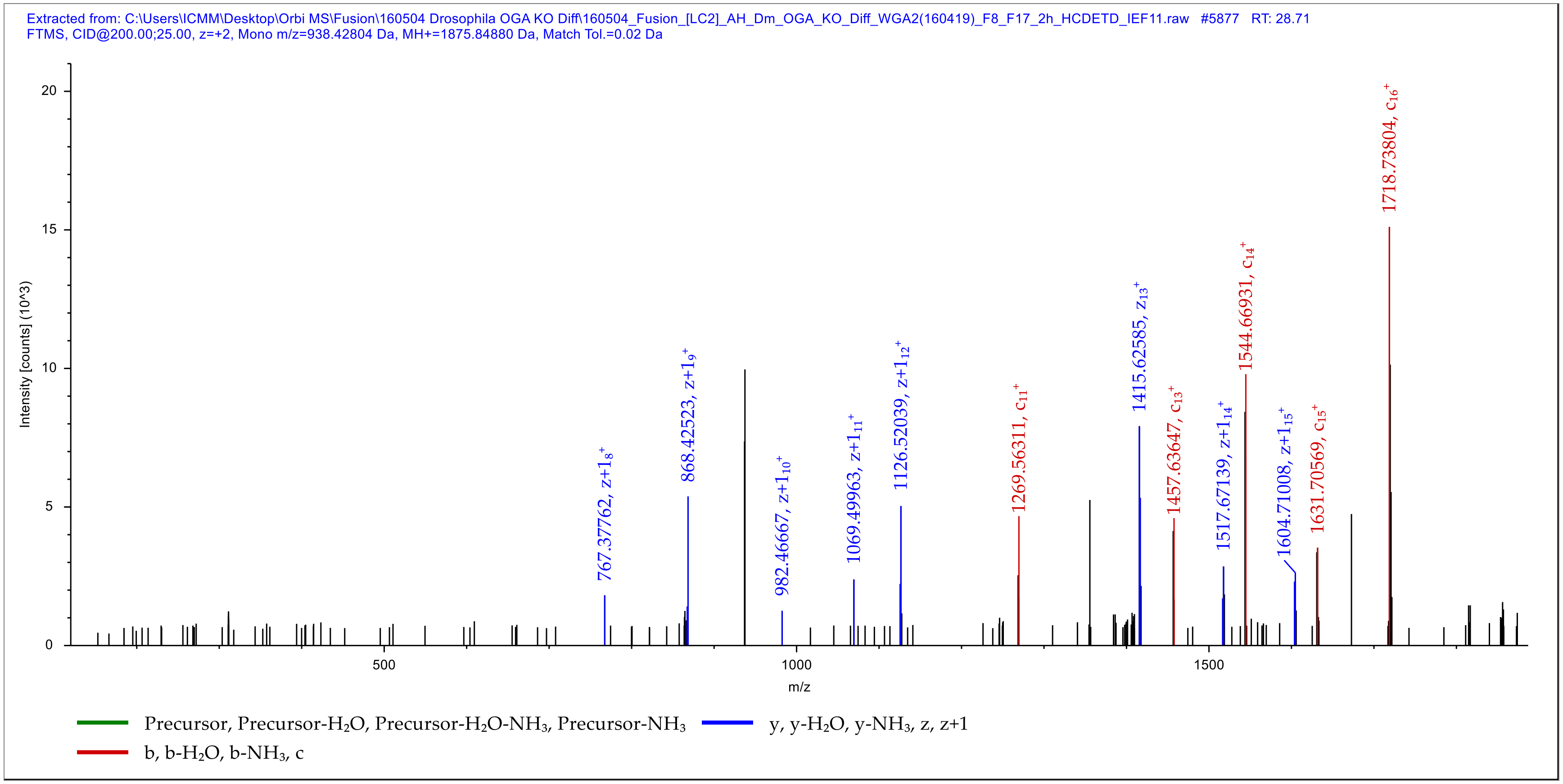

Extracted from: C:\Users\ICMM\Desktop\Orbi MS\Fusion\160504 Drosophila OGA KO Diff\160504_Fusion_[LC2]_AH_Dm_OGA_KO_Diff_WGA2(160419)_F8_F17_2h_HCDETD_IEF11.raw #5877 RT: 28.71
FTMS, CID@200.00;25.00, z=+2, Mono m/z=938.42804 Da, MH+=1875.84880 Da, Match Tol.=0.02 Da
Intensity [counts] (10^3)
m/z
767.37762, z+1$_8^+$
868.42523, z+1$_9^+$
982.46667, z+1$_{10}^+$
1069.49963, z+1$_{11}^+$
1126.52039, z+1$_{12}^+$
1269.56311, c$_{11}^+$
1415.62585, z$_{13}^+$
1457.63647, c$_{13}^+$
1517.67139, z+1$_{14}^+$
1544.66931, c$_{14}^+$
1604.71008, z+1$_{15}^+$
1631.70569, c$_{15}^+$
1718.73804, c$_{16}^+$
Precursor, Precursor-H$_2$O, Precursor-H$_2$O-NH$_3$, Precursor-NH$_3$
y, y-H$_2$O, y-NH$_3$, z, z+1
b, b-H$_2$O, b-NH$_3$, c

No. 3

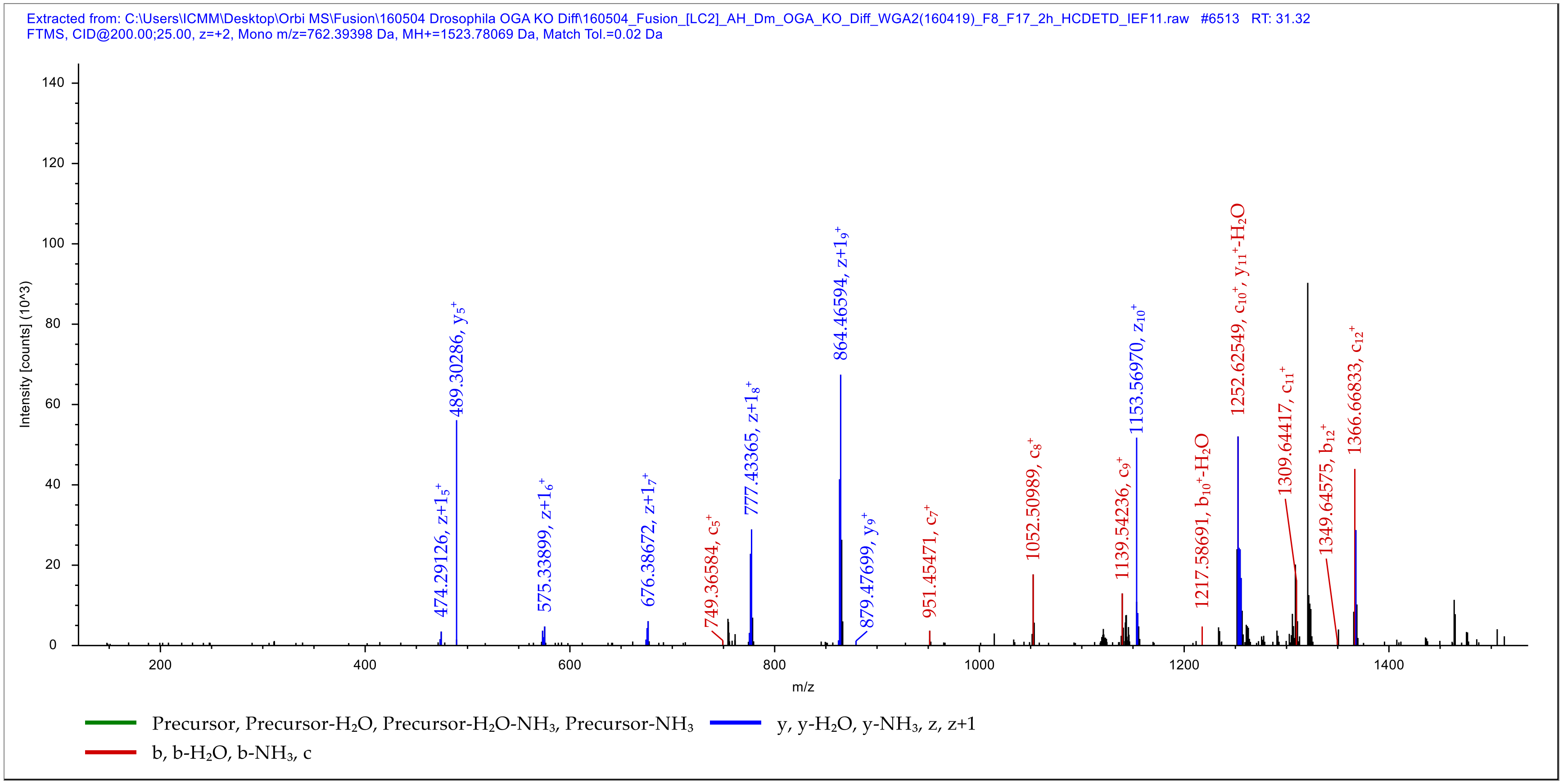

Extracted from: C:\Users\ICMM\Desktop\Orbi MS\Fusion\160504 Drosophila OGA KO Diff\160504_Fusion_[LC2]_AH_Dm_OGA_KO_Diff_WGA2(160419)_F8_F17_2h_HCDETD_IEF11.raw #6513 RT: 31.32
FTMS, CID@200.00;25.00, z=+2, Mono m/z=762.39398 Da, MH+=1523.78069 Da, Match Tol.=0.02 Da
Intensity [counts] (10^3)
m/z
474.29126, z+1₅⁺
489.30286, y₅⁺
575.33899, z+1₆⁺
676.38672, z+1₇⁺
749.36584, c₅⁺
777.43365, z+1₈⁺
864.46594, z+1₉⁺
879.47699, y₉⁺
951.45471, c₇⁺
1052.50989, c₈⁺
1139.54236, c₉⁺
1153.56970, z₁₀⁺
1217.58691, b₁₀⁺-H₂O
1252.62549, c₁₀⁺, y₁₁⁺-H₂O
1309.64417, c₁₁⁺
1349.64575, b₁₂⁺
1366.66833, c₁₂⁺
Precursor, Precursor-H₂O, Precursor-H₂O-NH₃, Precursor-NH₃
y, y-H₂O, y-NH₃, z, z+1
b, b-H₂O, b-NH₃, c

No. 4

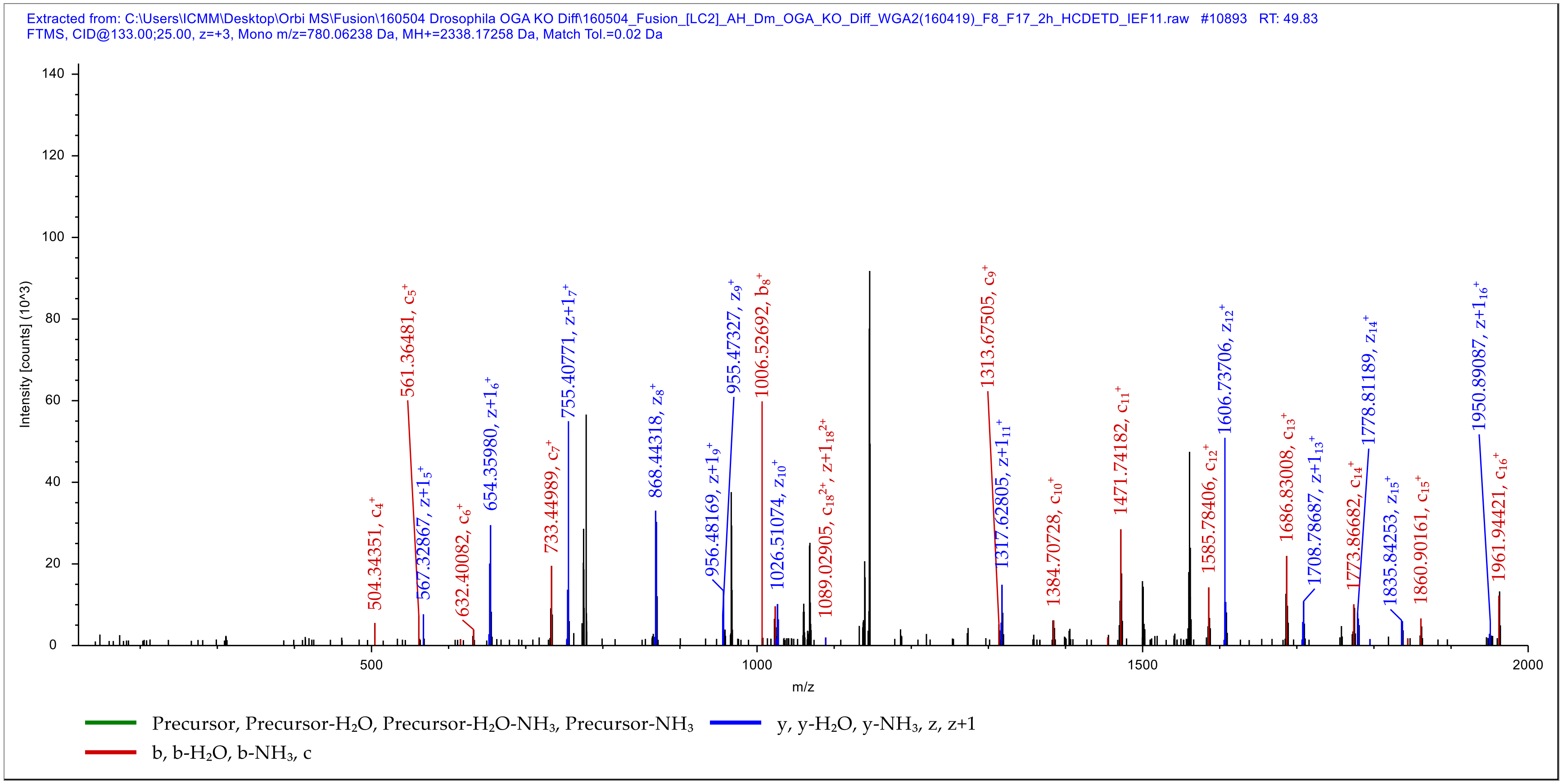

Extracted from: C:\Users\ICMM\Desktop\Orbi MS\Fusion\160504 Drosophila OGA KO Diff\160504_Fusion_[LC2]_AH_Dm_OGA_KO_Diff_WGA2(160419)_F8_F17_2h_HCDETD_IEF11.raw #10893 RT: 49.83
FTMS, CID@133.00;25.00, z=+3, Mono m/z=780.06238 Da, MH+=2338.17258 Da, Match Tol.=0.02 Da
Intensity [counts] (10^3)
m/z
504.34351, c4+
561.36481, c5+
567.32867, z+15+
632.40082, c6+
654.35980, z+16+
733.44989, c7+
755.40771, z+17+
868.44318, z8+
956.48169, z+19+
955.47327, z9+
1006.52692, b8+
1026.51074, z10+
1089.02905, c182+, z+1182+
1313.67505, c9+
1317.62805, z+111+
1384.70728, c10+
1471.74182, c11+
1585.78406, c12+
1606.73706, z12+
1686.83008, c13+
1708.78687, z+113+
1773.86682, c14+
1778.81189, z14+
1835.84253, z15+
1860.90161, c15+
1950.89087, z+116+
1961.94421, c16+
Precursor, Precursor-H2O, Precursor-H2O-NH3, Precursor-NH3
y, y-H2O, y-NH3, z, z+1
b, b-H2O, b-NH3, c

No. 5

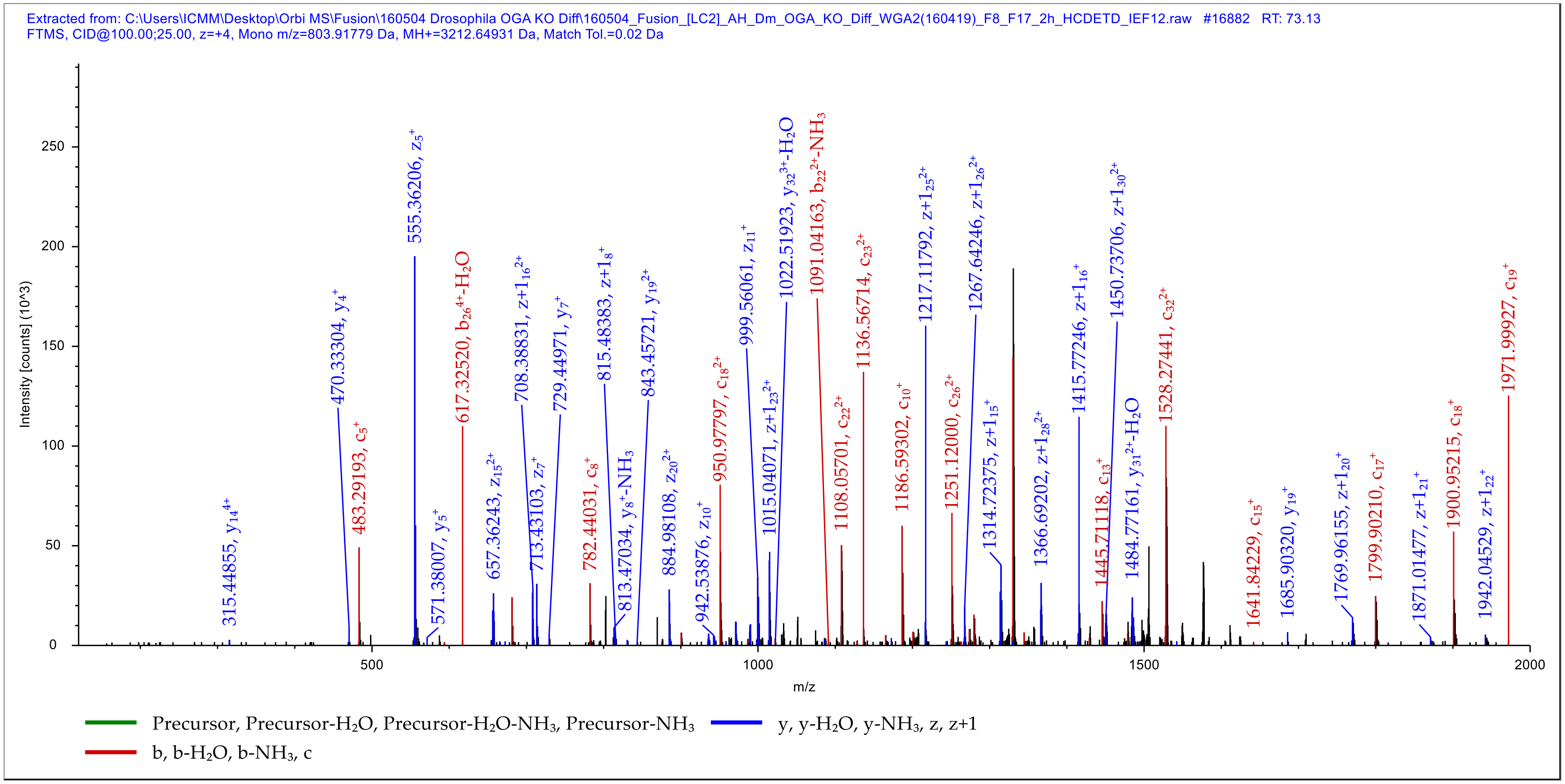
Extracted from: C:\Users\ICMM\Desktop\Orbi MS\Fusion\160504 Drosophila OGA KO Diff\160504_Fusion_[LC2]_AH_Dm_OGA_KO_Diff_WGA2(160419)_F8_F17_2h_HCDETD_IEF12.raw #16882 RT: 73.13
FTMS, CID@100.00;25.00, z=+4, Mono m/z=803.91779 Da, MH+=3212.64931 Da, Match Tol.=0.02 Da
Intensity [counts] (10^3)
m/z
315.44855, y_{14}^{4+}
470.33304, y_4^+
483.29193, c_5^+
555.36206, z_5^+
571.38007, y_5^+
617.32520, b_{26}^{4+}-H_2O
657.36243, z_{15}^{2+}
708.38831, $z{+}1_{16}^{2+}$
713.43103, z_7^+
729.44971, y_7^+
782.44031, c_8^+
813.47034, y_8^+-NH_3
815.48383, $z{+}1_8^+$
843.45721, y_{19}^{2+}
884.98108, z_{20}^{2+}
942.53876, z_{10}^+
950.97797, c_{18}^{2+}
999.56061, z_{11}^+
1015.04071, $z{+}1_{23}^{2+}$
1022.51923, y_{32}^{3+}-H_2O
1091.04163, b_{22}^{2+}-NH_3
1108.05701, c_{22}^{2+}
1136.56714, c_{23}^{2+}
1186.59302, c_{10}^+
1217.11792, $z{+}1_{25}^{2+}$
1251.12000, c_{26}^{2+}
1267.64246, $z{+}1_{26}^{2+}$
1314.72375, $z{+}1_{15}^+$
1366.69202, $z{+}1_{28}^{2+}$
1415.77246, $z{+}1_{16}^+$
1445.71118, c_{13}^+
1450.73706, $z{+}1_{30}^{2+}$
1484.77161, y_{31}^{2+}-H_2O
1528.27441, c_{32}^{2+}
1641.84229, c_{15}^+
1685.90320, y_{19}^+
1769.96155, $z{+}1_{20}^+$
1799.90210, c_{17}^+
1871.01477, $z{+}1_{21}^+$
1900.95215, c_{18}^+
1942.04529, $z{+}1_{22}^+$
1971.99927, c_{19}^+
Precursor, Precursor-H_2O, Precursor-H_2O-NH_3, Precursor-NH_3
y, y-H_2O, y-NH_3, z, z+1
b, b-H_2O, b-NH_3, c

No. 6

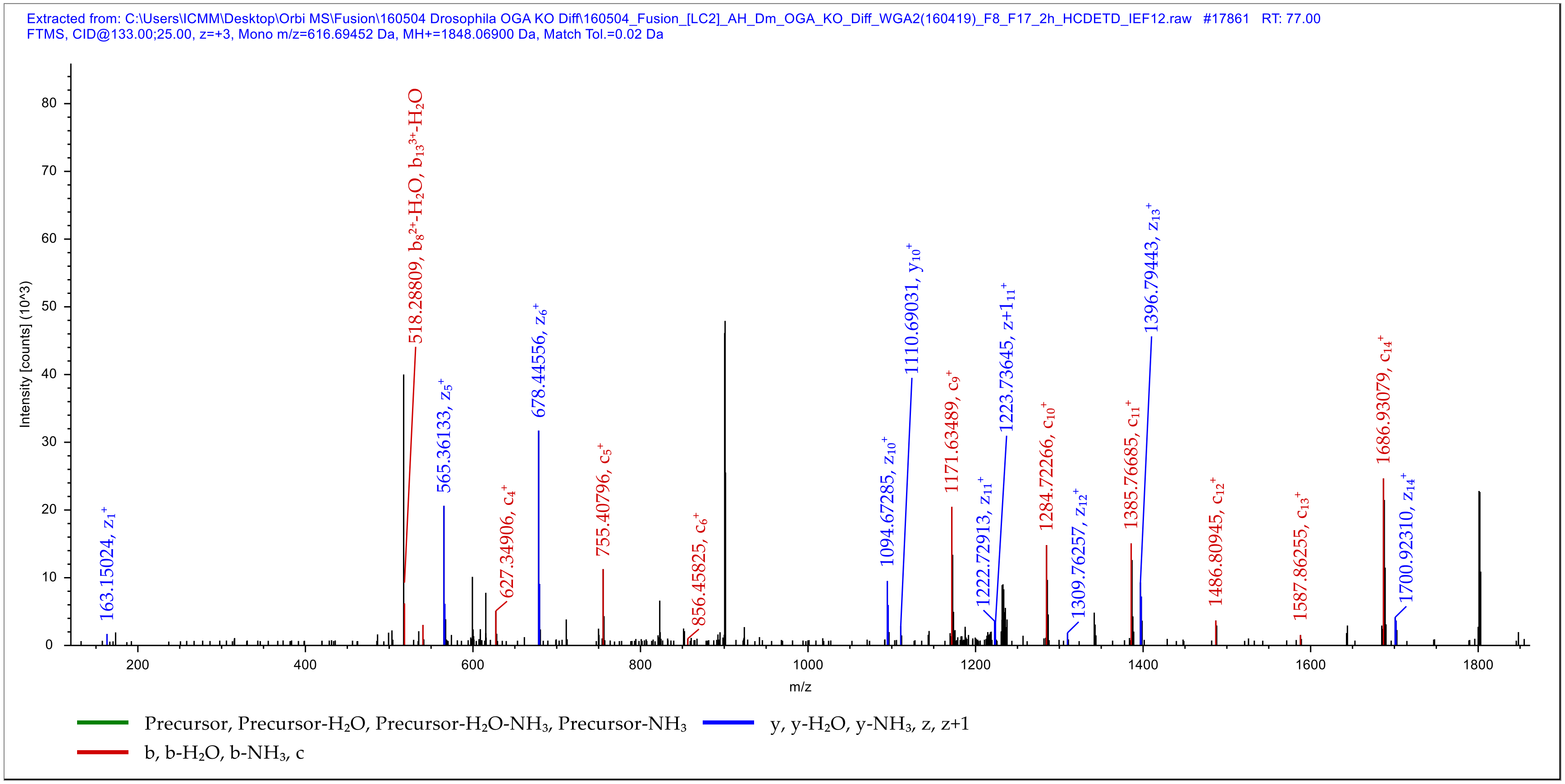

Extracted from: C:\Users\ICMM\Desktop\Orbi MS\Fusion\160504 Drosophila OGA KO Diff\160504_Fusion_[LC2]_AH_Dm_OGA_KO_Diff_WGA2(160419)_F8_F17_2h_HCDETD_IEF12.raw #17861 RT: 77.00
FTMS, CID@133.00;25.00, z=+3, Mono m/z=616.69452 Da, MH+=1848.06900 Da, Match Tol.=0.02 Da
Intensity [counts] (10^3)
m/z
163.15024, z_1^+
518.28809, b_8^{2+}-H_2O, b_{13}^{3+}-H_2O
565.36133, z_5^+
627.34906, c_4^+
678.44556, z_6^+
755.40796, c_5^+
856.45825, c_6^+
1094.67285, z_{10}^+
1110.69031, y_{10}^+
1171.63489, c_9^+
1222.72913, z_{11}^+
1223.73645, $z+1_{11}^+$
1284.72266, c_{10}^+
1309.76257, z_{12}^+
1385.76685, c_{11}^+
1396.79443, z_{13}^+
1486.80945, c_{12}^+
1587.86255, c_{13}^+
1686.93079, c_{14}^+
1700.92310, z_{14}^+
Precursor, Precursor-H_2O, Precursor-H_2O-NH_3, Precursor-NH_3
y, y-H_2O, y-NH_3, z, z+1
b, b-H_2O, b-NH_3, c

No. 7

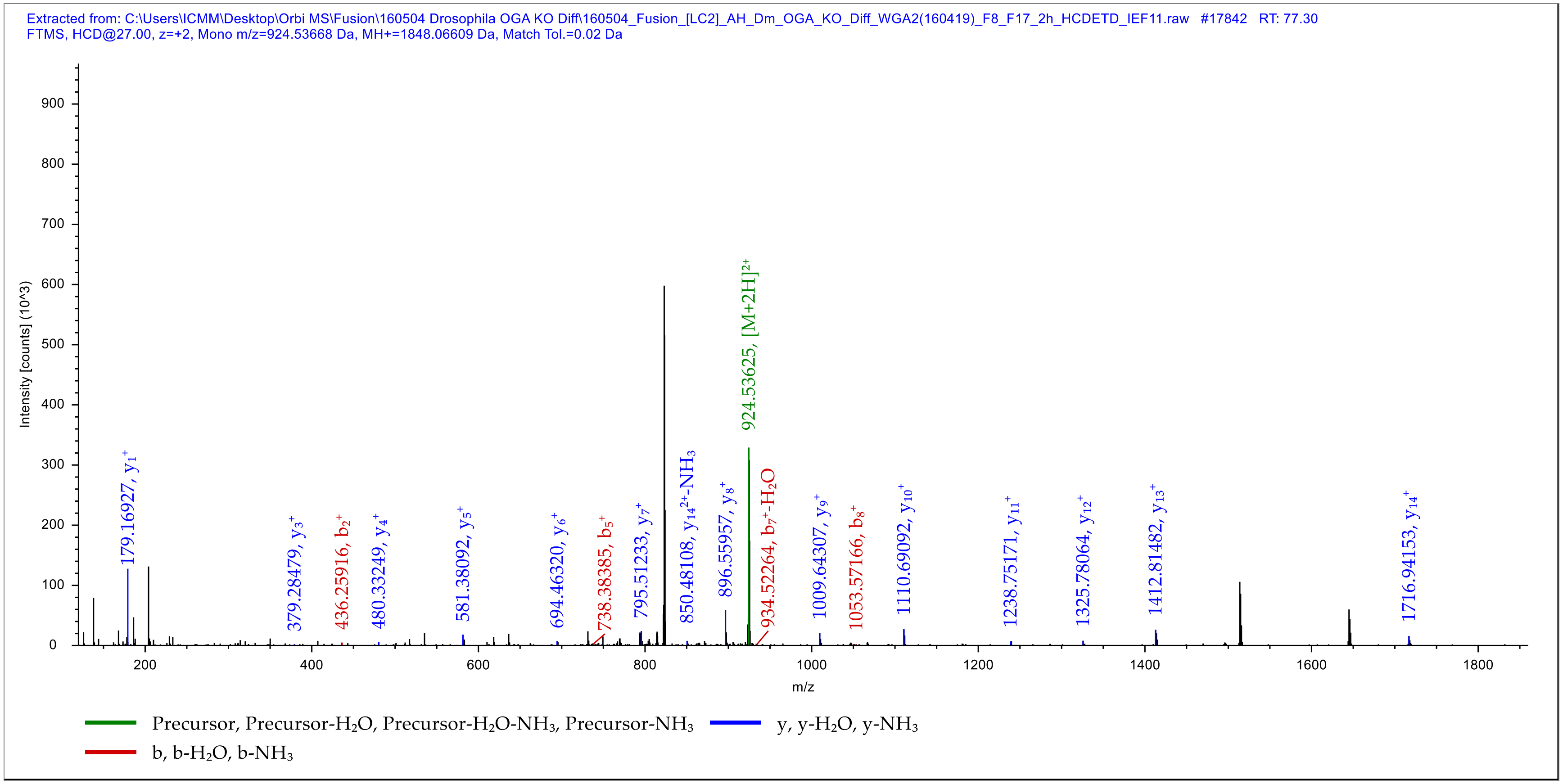

Extracted from: C:\Users\ICMM\Desktop\Orbi MS\Fusion\160504 Drosophila OGA KO Diff\160504_Fusion_[LC2]_AH_Dm_OGA_KO_Diff_WGA2(160419)_F8_F17_2h_HCDETD_IEF11.raw #17842 RT: 77.30
FTMS, HCD@27.00, z=+2, Mono m/z=924.53668 Da, MH+=1848.06609 Da, Match Tol.=0.02 Da
Intensity [counts] (10^3)
m/z
179.16927, y1+
379.28479, y3+
436.25916, b2+
480.33249, y4+
581.38092, y5+
694.46320, y6+
738.38385, b5+
795.51233, y7+
850.48108, y14 2+-NH3
896.55957, y8+
924.53625, [M+2H]2+
934.52264, b7+-H2O
1009.64307, y9+
1053.57166, b8+
1110.69092, y10+
1238.75171, y11+
1325.78064, y12+
1412.81482, y13+
1716.94153, y14+
Precursor, Precursor-H2O, Precursor-H2O-NH3, Precursor-NH3
y, y-H2O, y-NH3
b, b-H2O, b-NH3

No. 8

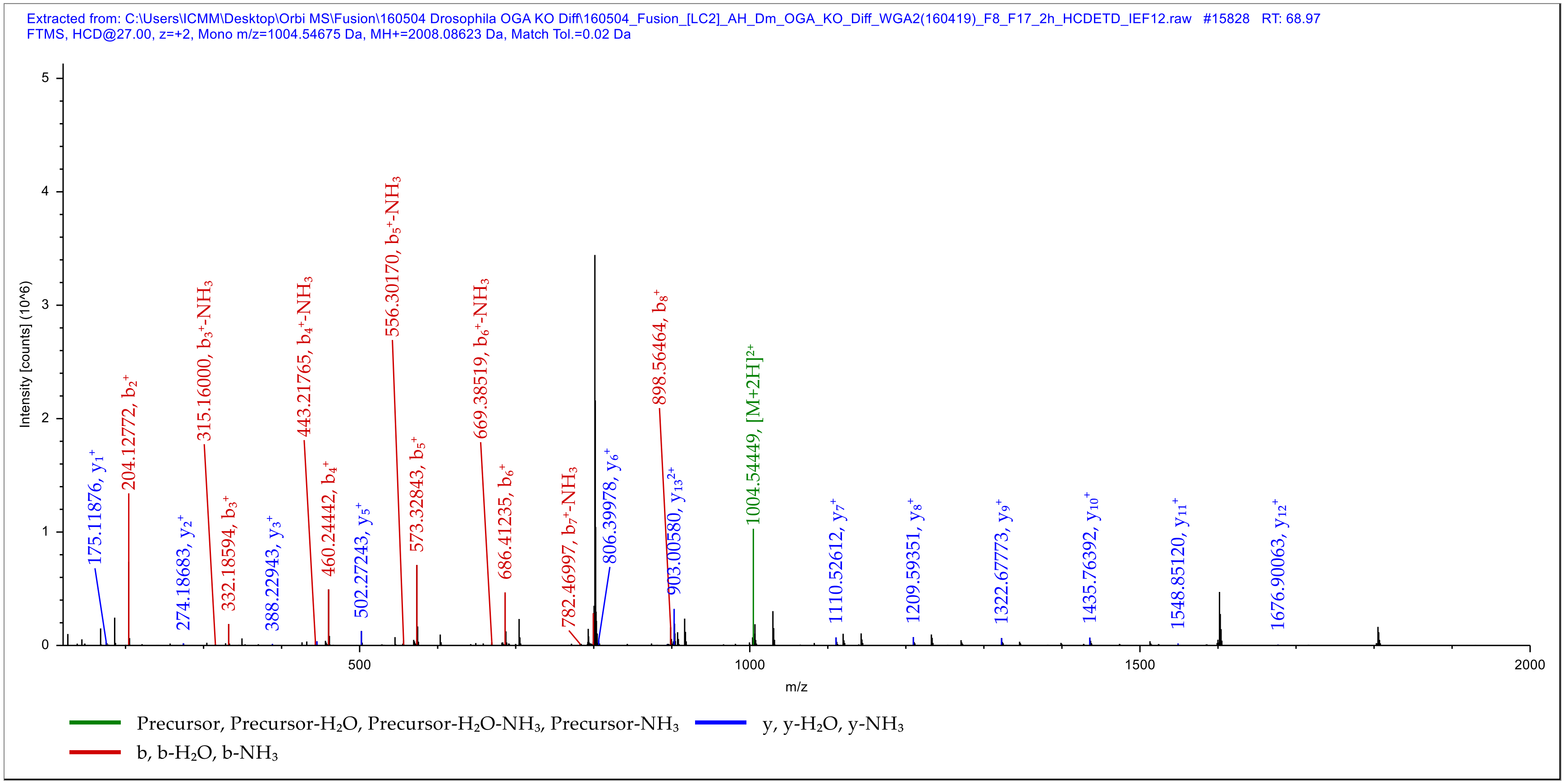

Extracted from: C:\Users\ICMM\Desktop\Orbi MS\Fusion\160504 Drosophila OGA KO Diff\160504_Fusion_[LC2]_AH_Dm_OGA_KO_Diff_WGA2(160419)_F8_F17_2h_HCDETD_IEF12.raw #15828 RT: 68.97
FTMS, HCD@27.00, z=+2, Mono m/z=1004.54675 Da, MH+=2008.08623 Da, Match Tol.=0.02 Da
Intensity [counts] (10^6)
m/z
175.11876, y_1^+
204.12772, b_2^+
274.18683, y_2^+
315.16000, b_3^+-NH_3
332.18594, b_3^+
388.22943, y_3^+
443.21765, b_4^+-NH_3
460.24442, b_4^+
502.27243, y_5^+
556.30170, b_5^+-NH_3
573.32843, b_5^+
669.38519, b_6^+-NH_3
686.41235, b_6^+
782.46997, b_7^+-NH_3
806.39978, y_6^+
898.56464, b_8^+
903.00580, y_{13}^{2+}
1004.54449, $[M+2H]^{2+}$
1110.52612, y_7^+
1209.59351, y_8^+
1322.67773, y_9^+
1435.76392, y_{10}^+
1548.85120, y_{11}^+
1676.90063, y_{12}^+
Precursor, Precursor-H_2O, Precursor-H_2O-NH_3, Precursor-NH_3
y, y-H_2O, y-NH_3
b, b-H_2O, b-NH_3

No. 9

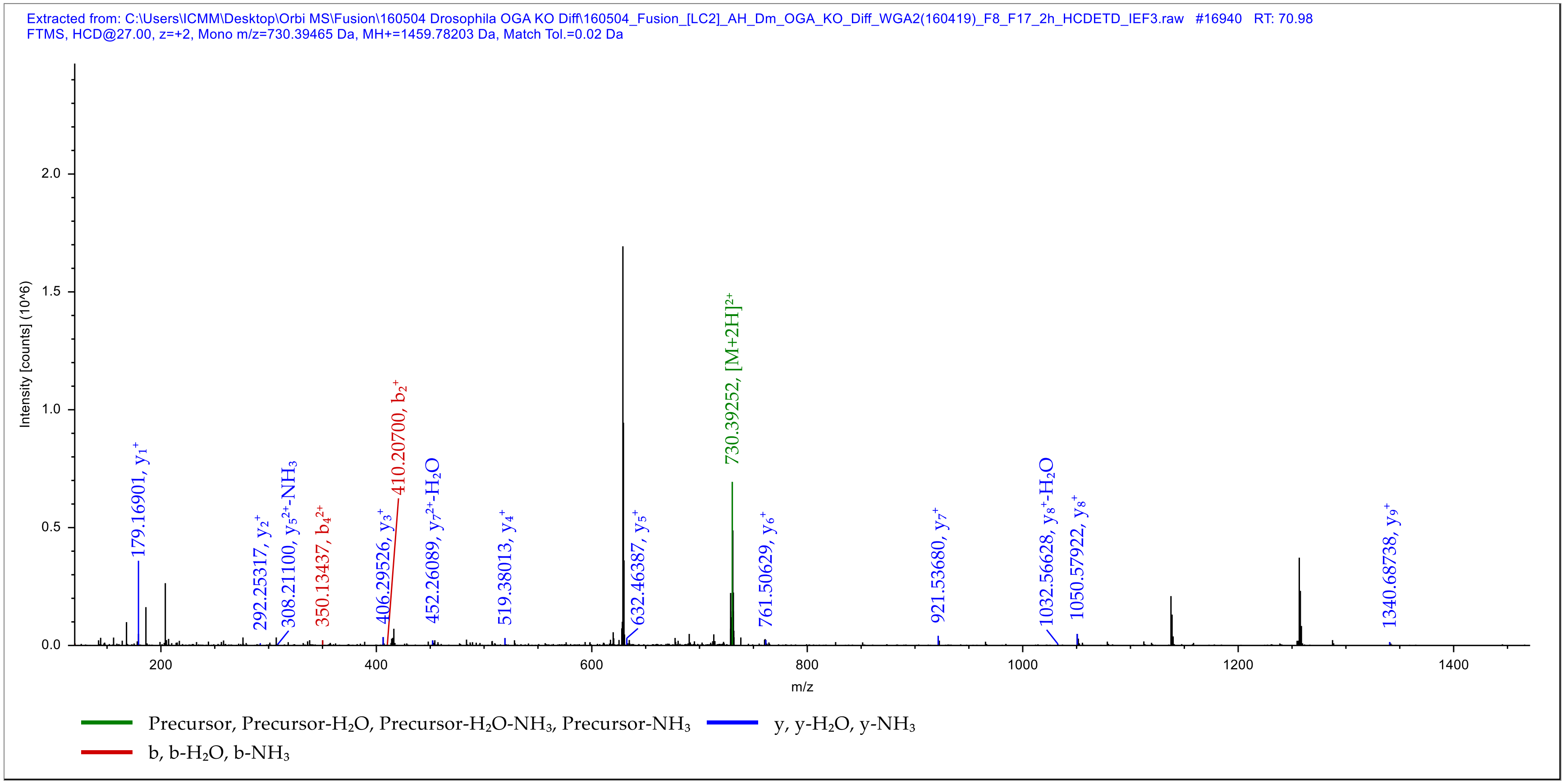

Extracted from: C:\Users\ICMM\Desktop\Orbi MS\Fusion\160504 Drosophila OGA KO Diff\160504_Fusion_[LC2]_AH_Dm_OGA_KO_Diff_WGA2(160419)_F8_F17_2h_HCDETD_IEF3.raw #16940 RT: 70.98
FTMS, HCD@27.00, z=+2, Mono m/z=730.39465 Da, MH+=1459.78203 Da, Match Tol.=0.02 Da
Intensity [counts] (10^6)
m/z
179.16901, y1+
292.25317, y2+
308.21100, y52+-NH3
350.13437, b42+
406.29526, y3+
410.20700, b2+
452.26089, y72+-H2O
519.38013, y4+
632.46387, y5+
730.39252, [M+2H]2+
761.50629, y6+
921.53680, y7+
1032.56628, y8+-H2O
1050.57922, y8+
1340.68738, y9+
Precursor, Precursor-H2O, Precursor-H2O-NH3, Precursor-NH3
y, y-H2O, y-NH3
b, b-H2O, b-NH3

No. 10

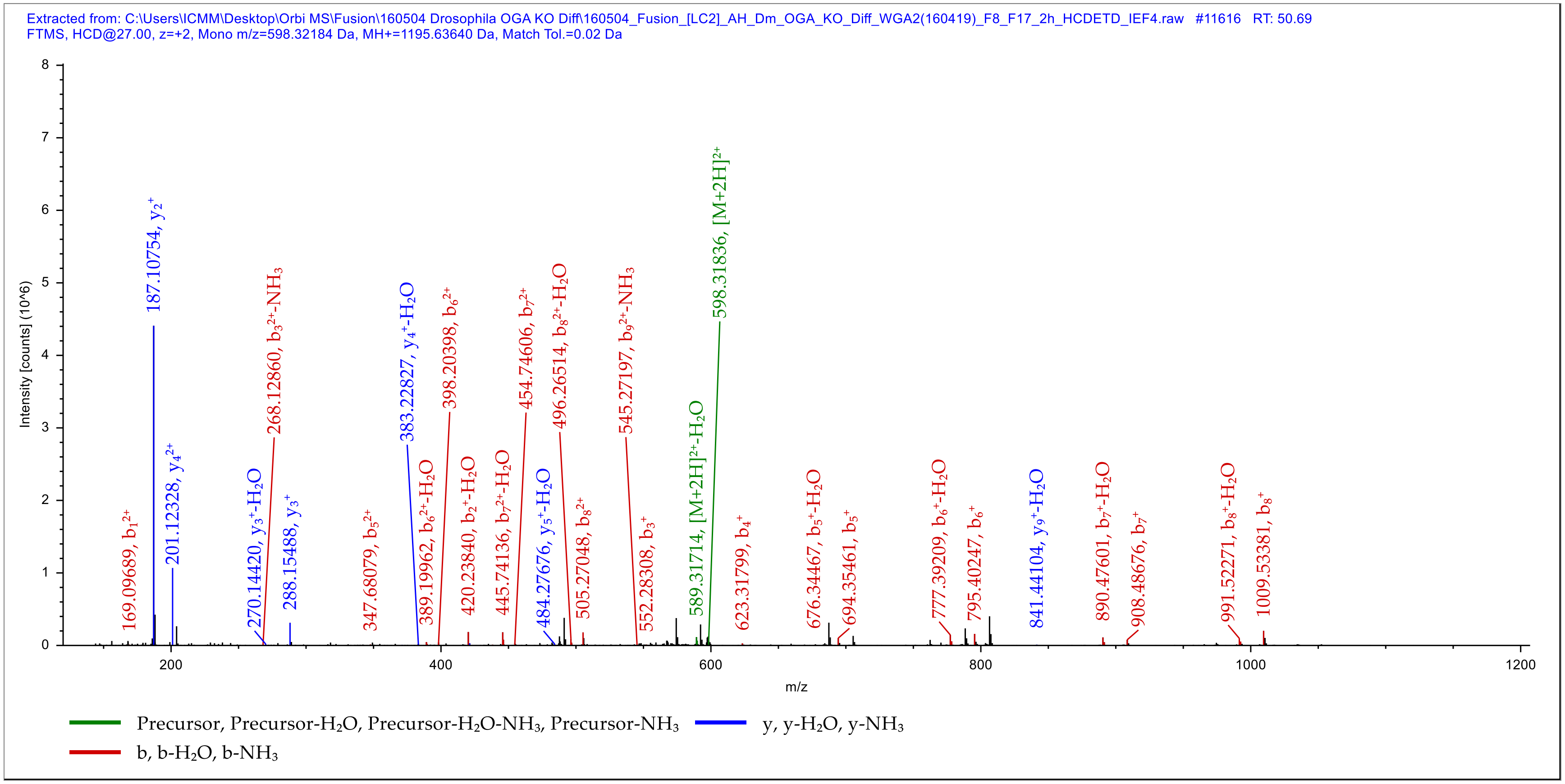
Extracted from: C:\Users\ICMM\Desktop\Orbi MS\Fusion\160504 Drosophila OGA KO Diff\160504_Fusion_[LC2]_AH_Dm_OGA_KO_Diff_WGA2(160419)_F8_F17_2h_HCDETD_IEF4.raw #11616 RT: 50.69
FTMS, HCD@27.00, z=+2, Mono m/z=598.32184 Da, MH+=1195.63640 Da, Match Tol.=0.02 Da
Intensity [counts] (10^6)
m/z
187.10754, y2+
201.12328, y4 2+
169.09689, b1 2+
270.14420, y3+-H2O
268.12860, b3 2+-NH3
288.15488, y3+
347.68079, b5 2+
383.22827, y4+-H2O
389.19962, b6 2+-H2O
398.20398, b6 2+
420.23840, b2+-H2O
445.74136, b7 2+-H2O
454.74606, b7 2+
484.27676, y5+-H2O
496.26514, b8 2+-H2O
505.27048, b8 2+
545.27197, b9 2+-NH3
552.28308, b3+
589.31714, [M+2H]2+-H2O
598.31836, [M+2H]2+
623.31799, b4+
676.34467, b5+-H2O
694.35461, b5+
777.39209, b6+-H2O
795.40247, b6+
841.44104, y9+-H2O
890.47601, b7+-H2O
908.48676, b7+
991.52271, b8+-H2O
1009.53381, b8+
Precursor, Precursor-H2O, Precursor-H2O-NH3, Precursor-NH3
y, y-H2O, y-NH3
b, b-H2O, b-NH3

No. 11

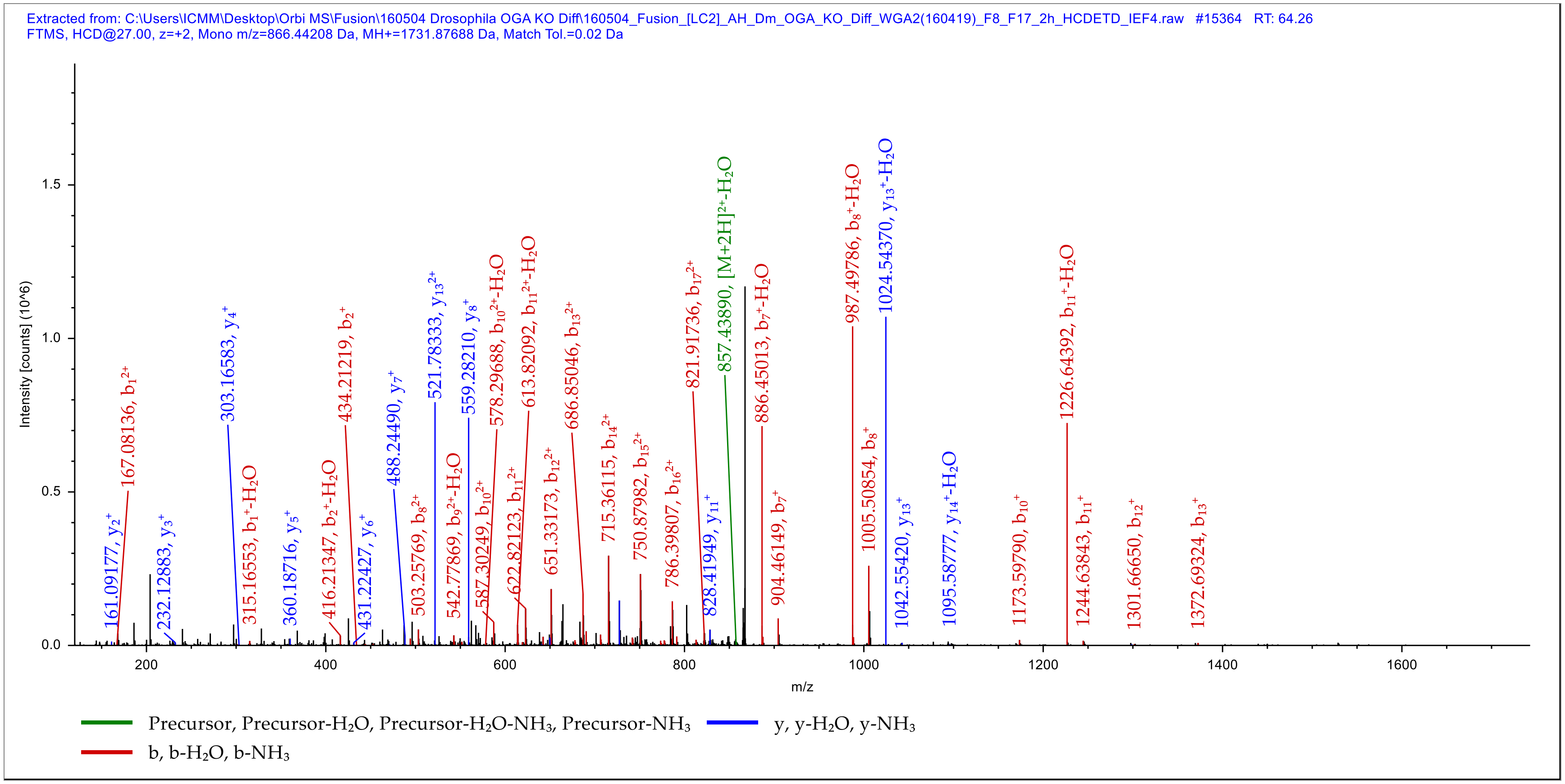

Extracted from: C:\Users\ICMM\Desktop\Orbi MS\Fusion\160504 Drosophila OGA KO Diff\160504_Fusion_[LC2]_AH_Dm_OGA_KO_Diff_WGA2(160419)_F8_F17_2h_HCDETD_IEF4.raw #15364 RT: 64.26
FTMS, HCD@27.00, z=+2, Mono m/z=866.44208 Da, MH+=1731.87688 Da, Match Tol.=0.02 Da
Intensity [counts] (10^6)
m/z
Precursor, Precursor-H2O, Precursor-H2O-NH3, Precursor-NH3
y, y-H2O, y-NH3
b, b-H2O, b-NH3

No. 12

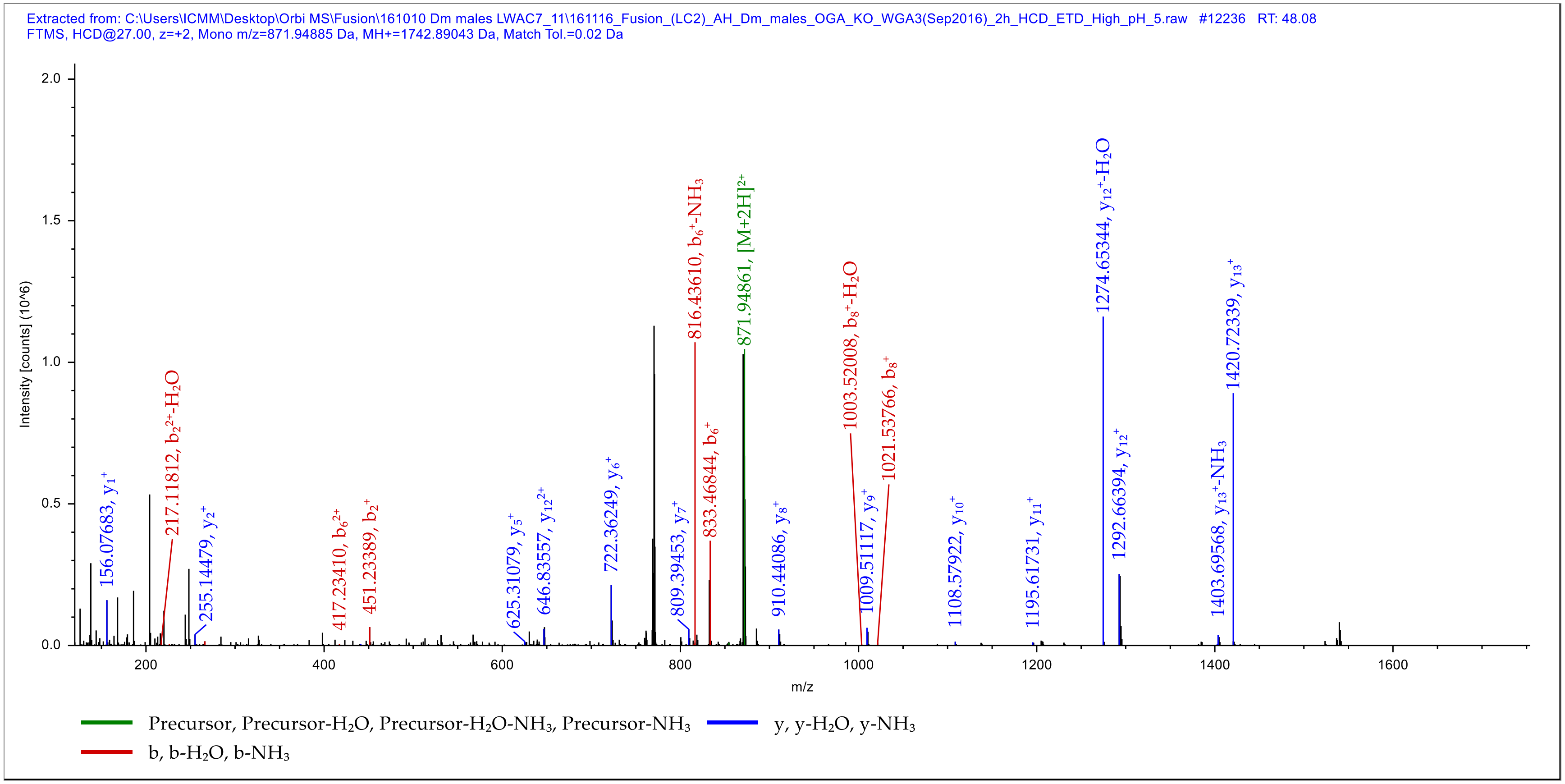
Extracted from: C:\Users\ICMM\Desktop\Orbi MS\Fusion\161010 Dm males LWAC7_11\161116_Fusion_(LC2)_AH_Dm_males_OGA_KO_WGA3(Sep2016)_2h_HCD_ETD_High_pH_5.raw #12236 RT: 48.08
FTMS, HCD@27.00, z=+2, Mono m/z=871.94885 Da, MH+=1742.89043 Da, Match Tol.=0.02 Da
Intensity [counts] (10^6)
m/z
156.07683, y1+
217.11812, b2 2+-H2O
255.14479, y2+
417.23410, b6 2+
451.23389, b2+
625.31079, y5+
646.83557, y12 2+
722.36249, y6+
809.39453, y7+
816.43610, b6+-NH3
833.46844, b6+
871.94861, [M+2H]2+
910.44086, y8+
1003.52008, b8+-H2O
1009.51117, y9+
1021.53766, b8+
1108.57922, y10+
1195.61731, y11+
1274.65344, y12+-H2O
1292.66394, y12+
1403.69568, y13+-NH3
1420.72339, y13+
Precursor, Precursor-H2O, Precursor-H2O-NH3, Precursor-NH3
y, y-H2O, y-NH3
b, b-H2O, b-NH3
